# Landau-Zener-Stückelberg Interferometry for Majorana Qubit

**DOI:** 10.1038/s41598-018-26324-5

**Published:** 2018-05-21

**Authors:** Zhi Wang, Wen-Chao Huang, Qi-Feng Liang, Xiao Hu

**Affiliations:** 1 School of Physics, Sun Yat-sen University, Guangzhou, 510275 China; 2 International Center for Materials Nanoarchitectonics (WPI-MANA), National Institute for Materials Science, Tsukuba, 305-0044 Japan; 3 Department of Physics, Shaoxing University, Shaoxing, 312000 China

## Abstract

Stimulated by a recent experiment observing successfully two superconducting states with even- and odd-number of electrons in a nanowire topological superconductor as expected from the existence of two end Majorana quasiparticles (MQs) [Albrecht *et al*., Nature **531**, 206 (2016)], we propose a way to manipulate Majorana qubit exploiting quantum tunneling effects. The prototype setup consists of two one-dimensional (1D) topological superconductors coupled by a tunneling junction which can be controlled by gate voltage. We show that the time evolution of superconducting phase difference at the junction under a voltage bias induces an oscillation in energy levels of the Majorana parity states, whereas the level-crossing is avoided by a small coupling energy of MQs in the individual 1D superconductors. This results in a Landau-Zener-Stückelberg (LZS) interference between the Majorana parity states. Adjusting pulses of bias voltage and gate voltage, one can construct a LZS interferometry which provides an arbitrary manipulation of the Majorana qubit.

## Introduction

Quasiparticle excitations in topological superconductors behave like Majorana fermions^1,2^. Considerable effort has been made to realize *zero-energy* Majorana quasiparticles (MQs) at ends of one-dimensional (1D) systems or vortex centers in 2D systems^3–21^, since these illusive quasiparticles obey the non-Abelian statistics which provides the basis for topological quantum computation^3,4,8,14,18^. Although the zero-energy MQs are charge neutral as they are constituted of electron and hole with equal weights, thus the equivalence between particle and antiparticle, two of them can compose a complex fermion, which specifies superconducting states carrying either odd- or even-number of electrons. Upon braiding the zero-energy MQs the superconducting system is driven from an eigenstate with a definite parity into superpositions of both parity states^3–5,8,12–15,18,19^, which can be exploited to build qubits stable against local electromagnetic noises. It is known, however, that the weights and phases of superposed Majorana qubit states cannot be controlled arbitrarily by braiding operations alone. For quantum computation, a universal gate enabling a complete control on Majorana qubits is required^22–30^.

In a recent experiment^31^, degenerate superconducting states with even- and odd-number of electrons in a nanowire topological superconductor of mesoscopic size have been revealed in terms of the Coulomb blockade effect^32,33^. This experimental breakthrough motivates us to seek for the possibility of building a compact universal gate for Majorana qubit in the nanowire system.

**Figure 1:**
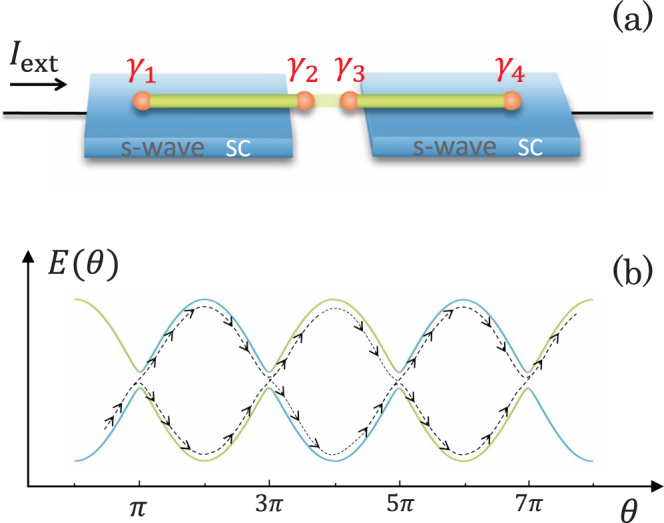
(**a**) Schematic of a LZS interferometry for Majorana qubit consisting of a quantum tunneling junction between two 1D topological superconductors driven by either voltage bias or current bias. (**b**) Energy levels of the two eigenstates as function of phase difference $E(\theta )=\pm \,\sqrt{{E}_{m}^{2}{\cos }^{2}(\theta \mathrm{/2)}+{\delta }^{2}}$ (see text), with the blue and green curves indicating the two pure parity states. The system in a parity eigenstate is scattered into a superposition of two parity states upon passing through $\theta =\mathrm{(2}n+\mathrm{1)}\pi$, and after a number of periods of oscillation in the superconducting phase difference evolves into the parity state opposite to the initial one as the manifestation of LZS interference.

We study a prototype setup of Majorana qubit involving a quantum tunneling junction between two 1D topological superconductors as sketched schematically in Fig. 1(a). The bias voltage across the junction drives the superconducting phase difference between the two superconductors to evolve with time according to the ac Josephson effect, and meanwhile generates small but appreciable interactions between MQs in individual segments. Solving the Schrödinger equation for the even- and odd-parity states of the Majorana qubit, we find intriguingly that the Majorana qubit rotates with time as a manifestation of Landau-Zener-Stückelberg (LZS) interference^34–37^, with a frequency proportional to the small MQs interactions in individual 1D topological superconductors. Furthermore, we demonstrate that one can control accurately the weights of the even- and odd-parity states by adjusting the pulse length of bias voltage, which, along with rotating the relative phase between the two parity states in terms of the gate voltage at the junction, achieves an arbitrary manipulation of the Majorana qubit. The present scheme is scalable, and the invasive disturbance to MQs is suppressed to the lowest level since the manipulation is based on quantum mechanical tunneling effects.

## Results

### Majorana quasiparticles in a 1D topological superconductor

The tight-binding Hamiltonian of a 1D semiconductor with strong spin-orbit coupling, proximity-induced superconducting gap and Zeeman energy, for example the left segment in Fig. 1a, is given by^38^

1 $$\begin{array}{rcl}H & = & -t\sum _{\langle i,j\rangle ,\sigma }{c}_{i\sigma }^{\dagger }{c}_{j\sigma }-(\mu +eV)\sum _{i,\sigma }{c}_{i\sigma }^{\dagger }{c}_{i\sigma }+\frac{\eta }{2}\sum _{i,\sigma ,\sigma ^{\prime} }{c}_{i+\mathrm{1,}\sigma }^{\dagger }{(i{\sigma }_{y})}_{\sigma \sigma ^{\prime} }{c}_{i,\sigma ^{\prime} }\\  &  & +\,\sum _{i,\sigma }{c}_{i,\sigma }^{\dagger }{({M}_{x}{\sigma }_{x})}_{\sigma \sigma ^{\prime} }{c}_{i,\sigma ^{\prime} }+\sum _{i}{\rm{\Delta }}{c}_{i,\uparrow }^{\dagger }{c}_{i,\downarrow }^{\dagger }+h.c.\end{array}$$

with *σ*_*x,z*_ the Pauli matrices, summation of *σ* on the eigenstates of *σ*_*z*_, *t* the nearest-neighbor hopping, *μ* the chemical potential which can be tuned by the voltage *V*, *η* the spin-orbit coupling, and *M*_*x*_ the Zeeman energy from the magnetic field. The superconducting gap Δ comes from an *s*-wave superconductor due to proximity effect, which therefore is *not* a result of mean-field approximation in conventional BdG approach. This Hamiltonian is casted into a matrix form $H=\tilde{c}\mathop{H}\limits^{ \sim }{\tilde{c}}^{\dagger }$ by introducing the particle-hole redundancy in terms of the Nambu vector $\tilde{c}=({c}_{\mathrm{1,}\uparrow }^{\dagger },\,{c}_{\mathrm{1,}\uparrow },\,{c}_{\mathrm{1,}\downarrow }^{\dagger },\,{c}_{\mathrm{1,}\downarrow },\,{c}_{\mathrm{2,}\uparrow }^{\dagger },\,\mathrm{...,}\,,\,\mathrm{...,}\,{c}_{N,\downarrow })$,

2 $$\mathop{H}\limits^{\sim }=\frac{1}{2}(\begin{array}{ccccccc}\cdot  & \cdot  & \cdot  & \cdot  & \cdot  & \cdot  & \cdot \\ \cdot  & {{\mathscr{H}}}_{0} & {{\mathscr{H}}}_{1} & 0 & 0 & 0 & \cdot \\ \cdot  & {{\mathscr{H}}}_{1}^{\ast } & {{\mathscr{H}}}_{0} & {{\mathscr{H}}}_{1} & 0 & 0 & \cdot \\ \cdot  & 0 & {{\mathscr{H}}}_{1}^{\ast } & {{\mathscr{H}}}_{0} & {{\mathscr{H}}}_{1} & 0 & \cdot \\ \cdot  & 0 & 0 & {{\mathscr{H}}}_{1}^{\ast } & {{\mathscr{H}}}_{0} & {{\mathscr{H}}}_{1} & \cdot \\ \cdot  & 0 & 0 & 0 & {{\mathscr{H}}}_{1}^{\ast } & {{\mathscr{H}}}_{0} & \cdot \\ \cdot  & \cdot  & \cdot  & \cdot  & \cdot  & \cdot  & \cdot \end{array})$$

with the on-site and nearest-neighbor matrices,

3 $${{\mathscr{H}}}_{0}=(\begin{array}{cccc}-\mu -eV & 0 & {M}_{x} & {\rm{\Delta }}\\ 0 & \mu +eV & -{{\rm{\Delta }}}^{\ast } & -{M}_{x}\\ {M}_{x} & -{\rm{\Delta }} & -\mu -eV & 0\\ {{\rm{\Delta }}}^{\ast } & -{M}_{x} & 0 & \mu +eV\end{array}),\,{{\mathscr{H}}}_{1}=(\begin{array}{cccc}-t & 0 & \eta /2 & 0\\ 0 & t & 0 & -\eta /2\\ -\eta /2 & 0 & -t & 0\\ 0 & \eta /2 & 0 & t\end{array}).$$

Diagonalizing Hamiltonian $\mathop{H}\limits^{ \sim }$ provides the eigenenergy *E*_*n*_ and the wavefunction ${\phi }_{n}(x)=$ $[{\mu }_{n,\uparrow }(x),{\nu }_{n,\uparrow }(x),{\mu }_{n,\downarrow }(x),{\nu }_{n,\downarrow }(x)]$. It is known that the system falls into a topological superconducting phase when the parameters satisfy the condition^9,10,38^ ${M}_{x}^{2} > (\mu -2+eV{)}^{2}+{{\rm{\Delta }}}^{2}$. The zero-energy wavefunction localized at the end of a semi-infinite system can be used to construct a creation operator,

4 $${\gamma }^{\dagger }=\int dx[{\mu }_{\mathrm{0,}\uparrow }(x){c}_{\uparrow }^{\dagger }(x)+{\nu }_{\mathrm{0,}\uparrow }(x){c}_{\uparrow }(x)+{\mu }_{\mathrm{0,}\downarrow }(x){c}_{\downarrow }^{\dagger }(x)+{\nu }_{\mathrm{0,}\downarrow }(x){c}_{\downarrow }(x)],$$

which defines a MQ as can be verified by inspecting ${\gamma }^{\dagger }=\gamma$ because ${\mu }_{\mathrm{0,}\sigma }={\nu }_{\mathrm{0,}\sigma }^{\ast }$ with $\sigma =\uparrow \,,\,\downarrow$.

**Figure 2:**
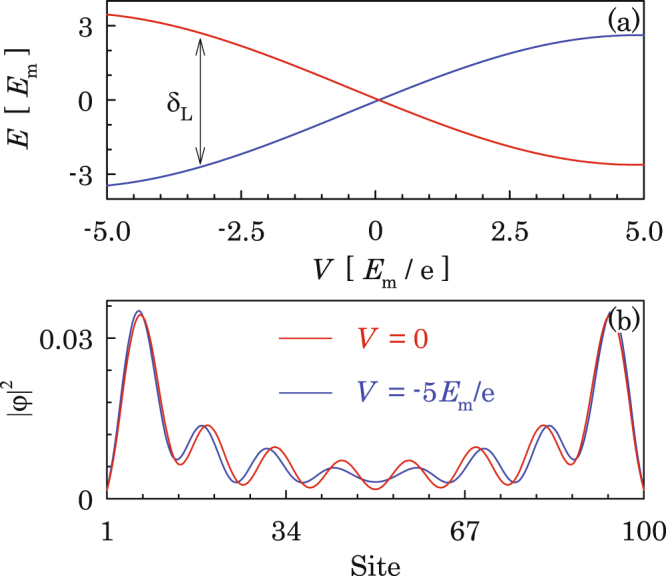
(**a**) Energy levels for the two parity states composed by the two MQs where the energy difference measures the coupling δ_L_ between the MQs, and (**b**) wavefunctions of the two MQs at *V* = 0 and $V=-\,5{E}_{{\rm{m}}}/e$ where the latter is intentionally taken large for clarity. The parameters are taken as $\eta =0.2t$, ${M}_{x}=0.04t$, ${\rm{\Delta }}=0.01t$, ${E}_{{\rm{m}}}=0.05{\rm{\Delta }}$ and $\mu =2t$.

The two energy levels within the superconducting gap for a finite 1D topological superconductor are displayed in Fig. 2a, where other quasiparticle excitations are omitted which carry high energies $E > {\rm{\Delta }}$. The energy difference between the two levels represents the coupling between the two MQs, which is determined by the overlapping between the wavefunctions of the two MQs as shown in Fig. 2b. For *V* = 0, the coupling between the two MQs is exponentially small, which grows rapidly by orders of magnitude when the voltage is applied due to the enhanced overlapping of the wavefunctions of MQs at the central part of the system. It is important to notice that, even with a finite coupling, the low-energy physics of the system is described by the two MQs, which define the odd- and even-parity of the system since other quasiparticle excitations are above the superconducting gap.

### LZS interference of Majorana parity states

As revealed by previous works^9,26^, the two MQs residing at the junction couple to each other in the form of $i{E}_{{\rm{m}}}{\gamma }_{2}{\gamma }_{3}$ through single-electron tunneling preocesses (see Fig. 1), where the energy *E*_*m*_ can be controlled by a gate voltage. At sufficiently low temperatures, currents flowing into and from the nanowires are carried by Cooper pairs, which preserves the total parity of the system. Therefore, the low-energy physics is described by the following Hamiltonian^39,40^,

5 $${H}_{{\rm{m}}}=-\,{E}_{{\rm{m}}}\,\cos (\theta \mathrm{/2)}{\tau }_{z}+\delta {\tau }_{x}$$

with basis of the even- and odd-parity state $i{\gamma }_{2}{\gamma }_{3}\mathrm{|0,}\,1\rangle =\pm \,\mathrm{|0,}\,1\rangle$, where *τ*_*x,z*_ are Pauli matrices and $\theta ={\varphi }_{1}-{\varphi }_{2}$ is the phase difference between the two superconductors which can be driven dynamically by a bias voltage across the junction. The interaction term *δ* between the two parity states is determined by couplings between MQs in individual 1D topological superconductors by $\delta ={\delta }_{L}+{\delta }_{R}$ [*δ*_*L*_ between *γ*_1_ and *γ*_2_ and *δ*_*R*_ between *γ*_3_ and *γ*_4_ as shown in Fig. 1(a)], which under an appropriate gauge can be put as a real number^41^. While being negligibly small at the pristine condition, *δ* is enhanced to an appreciable (but small) value when a bias voltage exists across the junction, as can be read from Fig. 2. For the purpose of manipulating the Majorana qubit, the junction is tuned by the gate voltage such that $\delta \ll {E}_{{\rm{m}}} < {\rm{\Delta }}$. In this situation, the eigenstates of the system are the superpositions of the two Majorana parity states around $\theta =\mathrm{(2}n+\mathrm{1)}\pi$ with the relative weights determined by the MQ interaction *δ*, whereas coincide with the two pure parity states elsewhere [see Eq. (5) and Fig. 1(b)].

When a finite voltage drop is induced across the junction, the superconducting phase difference starts to evolve with time according to the renowned ac Josephson relation, which triggers a quantum mechanical evolution of the Majorana qubit as can be read from Eq. (5). This dynamics can be described by the following the time-dependent Schrödinger equation,

6 $$i\hslash \frac{d}{dt}[\begin{array}{c}{\psi }_{0}\\ {\psi }_{1}\end{array}]=[\begin{array}{cc}-{E}_{{\rm{m}}}\,\cos \,\frac{\theta (t)}{2} & \delta \\ \delta  & {E}_{{\rm{m}}}\,\cos \,\frac{\theta (t)}{2}\end{array}]\,[\begin{array}{c}{\psi }_{0}\\ {\psi }_{1}\end{array}]$$

with ${\rm{\Psi }}={\psi }_{0}\mathrm{|0}\rangle +{\psi }_{1}\mathrm{|1}\rangle$ and $\theta (t)=2eVt/\hslash$ for a constant bias voltage^42,43^.

When the system is driven dynamically through $\theta =\mathrm{(2}n+\mathrm{1)}\pi$, the Majorana qubit may either stay in its original parity state, or evolve into the opposite parity state [see Fig. 1(b)]. The probability for staying in the same state is given by the ratio between the crossing-avoid energy and the velocity of the phase variation $\propto \,\exp [-{\delta }^{2}/(\hslash \omega {E}_{{\rm{m}}})]$ with $\omega =d\theta /dt$^34,35^. Experiencing many passages through the crossing points, the occupation probabilities of the two parity states are governed by the accumulation of quantum phases acquired at individual passages known as the LZS interference^34–37^, which yields a second, longer time scale as compared with the period of the oscillation of superconducting phase difference.

**Figure 3:**
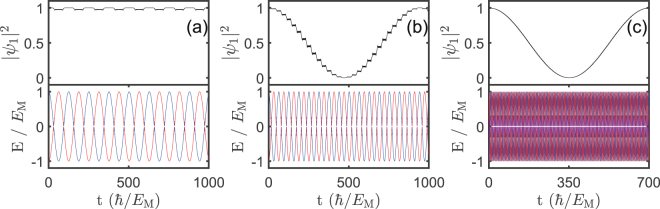
Time evolution of the Majorana qubit state given by $|{\psi }_{1}{|}^{2}$ (upper panel) and energies of the two qubit states (lower panel) for the bias voltage (**a**) $\omega =0.049955{E}_{{\rm{m}}}/\hslash$, (**b**) $\omega =0.1{E}_{{\rm{m}}}/\hslash$, and (**c**) $\omega ={E}_{{\rm{m}}}/\hslash$. Other parameters are taken same as Fig. 2.

The detailed quantum mechanical dynamics described by Eq. (6) can be revealed by numerical integration. As shown in Fig. 3, there is a typical LZS phenomenology with the interference pattern depending on the value of bias voltage. For very small bias voltages of $\omega \ll {E}_{{\rm{m}}}$ (see Fig. 3a), while LZ transitions occur at every avoided energy anti-crossing, the interference is destructive. For the bias voltage $\omega =0.1{E}_{{\rm{m}}}/\hslash$, while the interference becomes constructive (see Fig. 3b), the weight of $|{\psi }_{1}{|}^{2}$ experiences a series of step-like changes, which is not ideal for a smooth control of Majorana qubit either. For the bias voltages of $\omega ={E}_{{\rm{m}}}/\hslash$, which is still much smaller than the superconducting gap, the constructive LZS interference induces a smooth rotation in Majorana qubit as shown in Fig. 3c, which can be used for manipulation of the Majorana qubit.

**Figure 4:**
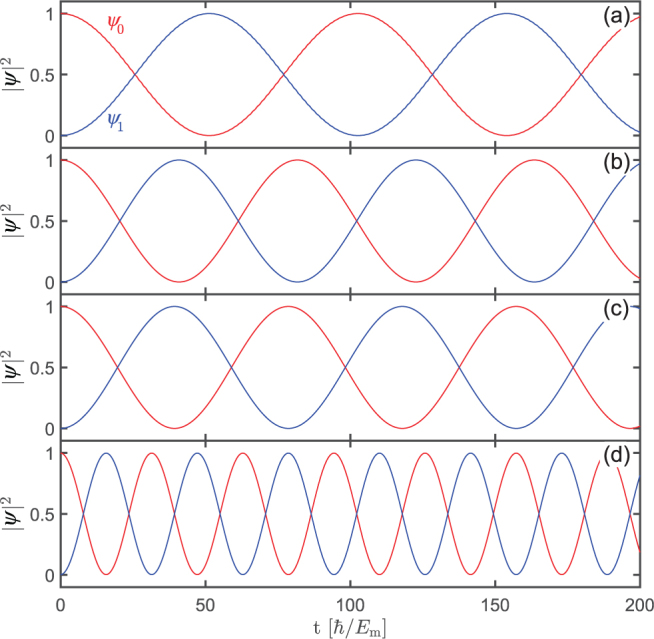
Simulation result for LZS interference between the two parity states with (**a**) $\omega =2{E}_{{\rm{m}}}/\hslash$, $\delta /{E}_{{\rm{m}}}=0.04$, (**b**) $\omega =5{E}_{{\rm{m}}}/\hslash$, $\delta /{E}_{{\rm{m}}}=0.04$, (**c**) $\omega =30{E}_{{\rm{m}}}/\hslash$, $\delta /{E}_{{\rm{m}}}=0.04$ and (**d**) $\omega =30{E}_{{\rm{m}}}/\hslash$, $\delta /{E}_{{\rm{m}}}=0.1$.

It is pedagogical to discuss the case with $\omega \gg {E}_{{\rm{m}}}$ for which an analytic description of the quantum mechanical evolution of the Majorana qubit is available resorting to the Floquet theorem^44,45^. For $\delta =0$ the time-dependent Schrödinger Eq. (6) can be transformed to the time-independent one with the Floquet matrix Hamiltonian of infinite dimensions corresponding to the photon numbers^46^. The effect of the small MQ interaction $\delta \ll {E}_{m},\omega$ can then be taken into account perturbatively. To the first-order perturbation approximation which involves a 2 × 2 Floquet matrix, we obtain a LZS oscillation in occupation probabilities $|{\psi }_{0}(t{)|}^{2}={\cos }^{2}({\omega }_{{\rm{m}}}t)$ and $|{\psi }_{1}(t{)|}^{2}={\sin }^{2}({\omega }_{{\rm{m}}}t)$ with

7 $${\omega }_{{\rm{m}}}=\delta {J}_{0}\mathrm{(4}{E}_{{\rm{m}}}/\hslash \omega )/\hslash ,$$

starting from the initial state ${\psi }_{0}\mathrm{(0)}=1$ and ${\psi }_{1}\mathrm{(0)}=0$, where *J*_0_(*x*) is the Bessel function (see Supplementary Materials). This analytical result can also be verified by numerical simulations. As shown in Fig. 4, the Majorana qubit rotates between the even- and odd-parity states for the three values of bias voltage. For bias voltages shown in Fig. 4(a,b), the frequency of qubit rotation depends on both bias voltage *ω* and coupling *δ*. For larger bias voltages shown in Fig. 4(c,d) the rotating frequency is proportional linearly to the coupling *δ*, independent of the bias voltage in agreement with Eq. (7). Explicitly, for the case of Fig. 4(c), Eq. (7) gives ${\omega }_{{\rm{m}}}=0.0398{E}_{{\rm{m}}}/\hslash$ taking into account $\omega =30{E}_{{\rm{m}}}/\hslash$, and numerical simulations give ${\omega }_{{\rm{m}}}=0.0399{E}_{{\rm{m}}}/\hslash$, whereas for the case of Fig. 4(d), one has ${\omega }_{{\rm{m}}}=0.0996{E}_{{\rm{m}}}/\hslash$ analytically and ${\omega }_{{\rm{m}}}=0.09955{E}_{{\rm{m}}}/\hslash$ numerically, in good agreement with each other in both cases. In contrary, for the case of Fig. 4(a), the analytical result gives ${\omega }_{{\rm{m}}}=0.00896{E}_{{\rm{m}}}/\hslash$, and numerical simulations give ${\omega }_{{\rm{m}}}=0.03055{E}_{{\rm{m}}}/\hslash$, and for the case of Fig. 4(b), one has ${\omega }_{{\rm{m}}}=0.03385{E}_{{\rm{m}}}/\hslash$ analytically and ${\omega }_{{\rm{m}}}=0.03835{E}_{{\rm{m}}}/\hslash$ numerically, with certain deviations between the two approaches since Eq. (7) is not applicable for small bias voltages. For the sake of simplicity, we will use large bias voltages for demonstration of the working principle of the LZS interferometry where the rotation of Majorana qubit is fast. One should keep in mind that the Hamiltonian in Eq. (5) may not be justified when the bias voltages is larger than the superconducting gap. Therefore, in practical implementation one should choose carefully the optimal bias voltage.

### LZS interferometry for Majorana qubit

**Figure 5:**
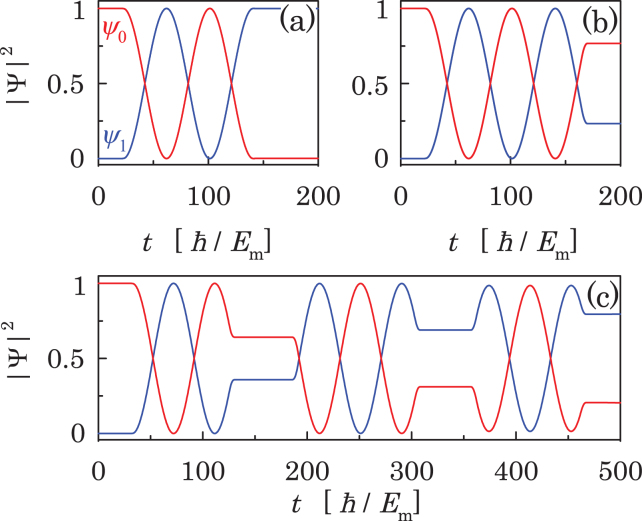
Simulation result for the LZS interferometry on the Majorana qubit based on pulse of bias voltage, with a short pulse (**a**) and a long pulse (**b**), and a sequence of three pulses (**c**). Parameters are the same as Fig. 4(a) except for *ω* = 0 and *δ* = 0 when the bias voltage is turned off.

Knowing that the Majorana qubit can make a full rotation between the even- and odd-parity states under sufficiently large bias voltages (but still below the superconducting gap), it is intriguing to check whether one can control the Majorana qubit arbitrarily. For this purpose, we investigate numerically the response of the system to pulses of bias voltage. As shown in Fig. 5(a,b), we observe that the occupation probabilities in the two Majorana qubit states start to oscillate when the bias voltage is turned on, and the oscillations stop when the bias voltage is turned off. Rotation between the two Majorana qubit states can either continue or switch back after turning on again the bias voltage, as seen in Fig. 5(c) at the second and third voltage pulse respectively, depending on both the state before the switching-off and time duration of switching-off. Therefore, weights of the even- and odd-parity states can be tuned arbitrarily when the length of voltage pulse is adjusted.

In order to manipulate the relative phase of the two Majorana qubit states, we can turn off the bias voltage yielding *δ* = 0, and meanwhile control the junction coupling *E*_m_ by lifting gate voltage to a sufficiently high value^26^ during designed time window [see Eq. (6)]. This completes an arbitrary control on the Majorana qubit. LZS interferometry has so far been proposed for manipulating superconducting flux qubits and Cooper-pair box qubits^37,46^. The present proposal is advantageous that only a bias voltage is required which can be performed quickly in contrast to operations involving magnetic flux.

**Figure 6:**
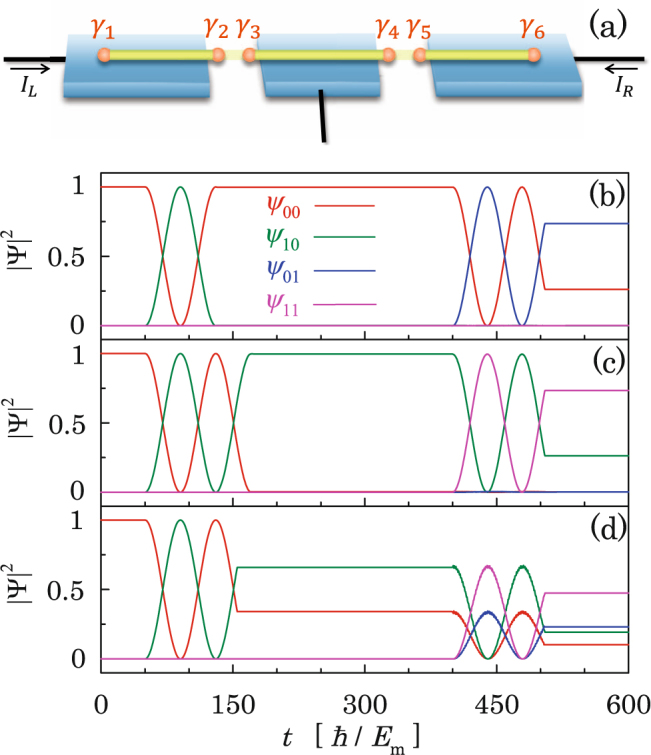
(**a**) Schematic for LZS interferometry for two Majorana qubits. (**b**–**d**) Simulation results for a sequence of controls on the left Majorana qubit with the right Majorana qubit staying static, and vice versa. In the first operation, the bias voltage in the left/right junction is switched on/off, and ${E}_{{\rm{m}},L}={E}_{{\rm{m}}}$ and ${E}_{{\rm{m}},{\rm{R}}}=2{E}_{{\rm{m}}}$ are taken; then bias voltage is switched off in both junctions for a time period during which the energies *E*_m,L_ and *E*_m,R_ are exchanged; in the second operation the bias voltage in the left/right junction is switched off/on. Parameters are the same as Fig. 5.

Because scalability is important for practical implementation of qubit gate, we investigate a system with two qubits in sequence as schematically shown in Fig. 6(a), where an electrode is attached to the superconductor at the middle such that bias voltage can be applied selectively at one of the two junctions. The time-dependent Schrödinger equation for the system of two Majorana qubits takes the same form as Eq. (6) except for that the Hamiltonian matrix is 4 × 4 with the diagonal entries ${\varepsilon }_{j}=\pm \,{E}_{{\rm{m}},{\rm{L}}}\,\cos ({\theta }_{{\rm{L}}}\mathrm{/2)}\pm {E}_{{\rm{m}},{\rm{R}}}\,\cos ({\theta }_{{\rm{R}}}\mathrm{/2)}$ standing for the energies of the four parity states with $j=\mathrm{|00}\rangle ,\,\mathrm{|10}\rangle ,\,\mathrm{|01}\rangle ,\,\mathrm{|11}\rangle$, where *E*_m,L_ and *E*_m,R_ are the two Majorana coupling constants at the left and right junctions respectively, and off-diagonal entries *δ*_*ij*_ standing for the couplings among the qubit states (see Supplement). Because the two Majorana *γ*_3_ and *γ*_4_ are residing on the same 1D topological superconductor [see Fig. 6(a)] and thus are intrinsically coupled to each other, at the first glance it would seem impossible to control the two Majorana qubits separately even though no bias voltage exists across one of the two junctions. A careful analysis gives a positive answer since a Majorana qubit changes its parity only when the two parity states are close to each other in energy as compared with the MQ interaction *δ* as can be seen in Fig. 1(b). We therefore assign the two coupling energies at the junctions to satisfy $|{E}_{{\rm{m}},{\rm{L}}}-{E}_{{\rm{m}},{\rm{R}}}|\gg \delta$. We then look at the time evolution of the wavefunction $|\psi \rangle ={\psi }_{00}\mathrm{|00}\rangle +{\psi }_{01}\mathrm{|01}\rangle +{\psi }_{10}\mathrm{|10}\rangle +{\psi }_{11}\mathrm{|11}\rangle$ under several typical pulse operations. As shown in Fig. 6(b,c), in the first operation the left Majorana qubit rotates with the right one remaining static, and vice versa in the second operation; one can stop the left Majorana qubit at either the even-parity state [Fig. 6(b)] or the odd-parity state [Fig. 6(c)]. One can also choose to stop the left Majorana qubit at a superposed state of the even- and odd-parity states as shown in Fig. 6(d) in the second pulse of bias voltage where both $|{\psi }_{00}{|}^{2}+|{\psi }_{01}{|}^{2}$ and $|{\psi }_{10}{|}^{2}+|{\psi }_{11}{|}^{2}$ remain constant. This observation indicates unambiguously that the weights of parity states at the two Majorana qubits can be controlled independently. In a long sequence, flipping the parity of a given Majorana qubit involves high-order quantum processes, which takes longer operation time.

## Discussions

In the present approach, we concentrate on the quantum mechanical dynamics of the Majorana qubit formed by the two MQs at the junction between two nanowires, taking into account the fact that the parity of the whole system of four MQs is conserved. Rotation of the Majorana qubit is accompanied by single-electron transportation from the left to right nanowire via the junction. In this regard, we notice that dynamics of MQs in a single nanowire was addressed explicitly in a recent work under a sinusoidal time-dependent voltage introduced by gates at the nanowire ends^47^.

To summarize, we reveal theoretically a Landau-Zener-Stückelberg interference of Majorana parity states in a junction between two one-dimensional topological superconductors under a bias voltage. We demonstrate that the Majorana qubit can be rotated completely between the even- and odd-parity states, and a Landau-Zener-Stückelberg interferometry can be implemented by adjusting the pulse length of bias voltage, which along with the control of junction with gate voltage provides an arbitrary manipulation of the Majorana qubit. The invasive disturbance to Majorana quasiparticles is suppressed to the lowest level since the manipulation is totally based on quantum mechanical tunneling effects, and the scheme is scalable.

## Methods

We solve numerically the Bogoliubov de-Gennes equation for a tight-binding Haimltonian of nanowire with the spin-orbit coupling, the Zeeman energy, and the proximity induced superconducting gap, and obtain the Majorana quasiparticles residing at the two ends of the nanowire, with very small energies caused by mutual coupling as a function of the chemical potential. Then we consider two such nanowires coupled with each other with a weak junction. In terms of the standard Runge-Kutta method, we analyze the dynamics under bias voltages between the two nanowires by solving numerically the Schrödinger equation on an effective two-level problem described explicitly by the two Majorana quaispartcles at the junction, which manifests a Landau-Zener-Stückelberg interference between the two Majorana qubit states. We also derive analytically the frequency of the Landau-Zener-Stückelberg interference as a function of bias voltage in terms of the Floquet theory.

## Electronic supplementary material

Supplemetary Materials
