# Supplementary material for: Landau-Zener-Stückelberg Interferometry for Majorana Qubit
Source: Sci Rep. 2018 May 21;8:7920. doi: 10.1038/s41598-018-26324-5 (PMC5962612; doi:10.1038/s41598-018-26324-5)
Supplement: Supplementary file 1 — Supplemetary Materials [file 41598_2018_26324_MOESM1_ESM.pdf]

Supplementary Materials for "Landau-Zener-Stückelberg Interferometry for Majorana Qubit"

Zhi Wang, Wen-Chao Huang, Qi-Feng Liang and Xiao Hu

Floquet theory for Majorana qubit. Here we show that the quantum oscillation of the Majorana qubit due to the Landau-Zener-Stückelberg (LZS) interference can be derived analytically in the lowest-order approximation. Under a constant bias voltage, the superconducting phase difference across the junction between two 1D topological superconductors (see Fig. 1 in the main text) evolves with time linearly. In this case the time-dependent Schrödinger equation for the Majorana qubit states reads

$$i\hbar\frac{d}{dt}\begin{pmatrix}\psi_0\\ \psi_1\end{pmatrix}=\begin{pmatrix}-E_{\rm m}\cos\frac{\omega t}{2} & \delta\\ \delta & E_{\rm m}\cos\frac{\omega t}{2}\end{pmatrix}\begin{pmatrix}\psi_0\\ \psi_1\end{pmatrix} \tag{S1}$$

as given in the main text. Because the Hamiltonian is time periodic, Eq. (S1) can be tackled in terms of the Floquet theory. A previous study[1] shows that, up to the lowest-order perturbation the time evolution of the quantum state is governed by the following effective Floquet Hamiltonian,

$$H_{\rm F}=\begin{pmatrix}0 & \delta J_0(2z)\\ \delta J_0(2z) & 0\end{pmatrix}, \tag{S2}$$

where $z = 2E_{\rm m}/\omega\hbar$ and J_0 is the Bessel function. The evolution operator for this effective Floquet Hamiltonian is,

$$\hat{U}(t)=e^{-i\hat{H}_F t/\hbar}=e^{-i\delta J_0(2z)\sigma_x t/\hbar}, \tag{S3}$$

which gives an oscillation of the form,

$$|\psi_0(t)|^2=\cos^2(\omega_{\rm m}t),\qquad |\psi_1(t)|^2=\sin^2(\omega_{\rm m}t). \tag{S4}$$

starting from an initial state $\psi_1 = 0$ with,

$$\omega_{\rm m}=\delta J_0(2z)/\hbar. \tag{S5}$$

This is the LZS interference in the Majorana qubit addressed in the main text.

Hamiltonian for Two Qubits. In this section we derive the Hamiltonian for the quantum mechanical dynamics of two Majorana qubits in a matrix form. The system consists of two tunneling junctions between three 1D topological superconductors as illustrated in Fig. 6(a) in the main text. The Hamiltonian is given as,

$$\begin{aligned} H_{\mathrm{m}} = & \, i\gamma_2\gamma_3 E_{\mathrm{m,L}} \cos\frac{\theta_{\mathrm{L}}}{2} + i\gamma_4\gamma_5 E_{\mathrm{m,R}} \cos\frac{\theta_{\mathrm{R}}}{2} \\ & + i\delta_1\gamma_1\gamma_2 + i\delta_2\gamma_3\gamma_4 + i\delta_3\gamma_5\gamma_6, \end{aligned} \tag{S6}$$

where $\theta_{\mathrm{L}} = \phi_2 - \phi_1$ and $\theta_{\mathrm{R}} = \phi_3 - \phi_2$ is the phase difference, $E_{\mathrm{m,L}}$ and $E_{\mathrm{m,R}}$ is the coupling energy of MQs at the left and right junction respectively, and $\delta_{1,2,3}$ are the coupling energies due to the wavefunction overlappings of MQs in the three topological superconductors. In terms of the six MQs one can define three complex fermions,

$$f_1^\dagger = \frac{(\gamma_2 + i\gamma_3)}{2}, \quad f_2^\dagger = \frac{(\gamma_4 + i\gamma_5)}{2}, \quad f_3^\dagger = \frac{(\gamma_6 + i\gamma_1)}{2}. \tag{S7}$$

Then Hamiltonian (S6) can be transformed into the fermionic form,

$$\begin{aligned} H_{\mathrm{m}} = & (1 - 2f_1^\dagger f_1) E_{\mathrm{m,L}} \cos\frac{\theta_{\mathrm{L}}}{2} + (1 - 2f_2^\dagger f_2) E_{\mathrm{m,R}} \cos\frac{\theta_{\mathrm{R}}}{2} \\ & + \delta_1 (f_3^\dagger f_1 + f_3^\dagger f_1^\dagger - f_3 f_1 - f_3 f_1^\dagger) \\ & + \delta_2 (f_1^\dagger f_2 + f_1^\dagger f_2^\dagger - f_1 f_2 - f_1 f_2^\dagger) \\ & + \delta_3 (f_2^\dagger f_3 + f_2^\dagger f_3^\dagger - f_2 f_3 - f_2 f_3^\dagger). \end{aligned} \tag{S8}$$

Because the total parity of the system is conserved, one can concentrate on the even-parity subspace without losing generality. In this subspace, we can take basis states, $|00\rangle = |0\rangle$, $|10\rangle = f_1^\dagger f_3^\dagger |0\rangle$, $|01\rangle = f_2^\dagger f_3^\dagger |0\rangle$, and $|11\rangle = f_1^\dagger f_2^\dagger |0\rangle$, with $|0\rangle$ the vacuum state. Then the Hamiltonian is rewritten in a matrix form

$$H_m = \begin{pmatrix} E_L + E_R & -\delta_1 & \delta_3 & \delta_2 \\ -\delta_1 & -E_L + E_R & \delta_2 & \delta_3 \\ \delta_3 & \delta_2 & E_L - E_R & \delta_1 \\ \delta_2 & \delta_3 & \delta_1 & -E_L - E_R \end{pmatrix}, \tag{S9}$$

with $E_L = E_{\mathrm{m,L}} \cos\frac{\theta_{\mathrm{L}}}{2}$ and $E_R = E_{\mathrm{m,R}} \cos\frac{\theta_{\mathrm{R}}}{2}$.

The superconducting phase differences of the two junctions θ_{L} and θ_{R} are controlled by the two bias voltages. The coupling energies $E_{\mathrm{m,L}}$ and $E_{\mathrm{m,R}}$ can be tuned by gate voltages.

Solving the Schrödinger equation with the matrix Hamiltonian presuming the linear time-dependence of $\theta_{\rm L}$ and $\theta_{\rm R}$ and $\delta_1 = \delta_2 = \delta_3 = \delta$ we obtain the results displayed in Fig. 6 in the main text.

RCSJ phase dynamics. The LZS interferometry for Majorana qubit can also be achieved under current bias. In order to show this explicitly, we analyze the resistively-and-capacitively-shunted dynamics of superconducting phase difference at the junction[2,3]

$$I_{\rm ext} = \frac{\hbar C\ddot{\theta}}{2e} + \frac{\hbar\dot{\theta}}{2eR} + I_{\rm s}\sin\theta + I_{\rm m}(|\psi_0|^2 - |\psi_1|^2)\sin\frac{\theta}{2}, \tag{S10}$$

where C and R are the effective capacitance and resistance of the junction [see Fig. 1(a) in the main text], and $I_{\rm s}$ and $I_{\rm m}$ are the threshold currents for the channels of Cooper pairs and MQs respectively, with the last term proportional to $\langle i\gamma_2\gamma_3\rangle$[4,5]. This semi-classical treatment on superconducting phase difference is possible since it evolves with time much faster than the Majorana qubit [see Eq. (7) in the main text].

The tunneling junction has both conventional Cooper-pair and MQ channels, and the total supercurrent depends on the superconducting phase difference in a way given by

$$I(\theta) = I_{\rm s}\sin\theta + I_{\rm m}(|\psi_0|^2 - |\psi_1|^2)\sin\frac{\theta}{2}. \tag{S11}$$

We observe that, in a static state, the Majorana parity state with $|\psi_0| = 1$ and $|\psi_1| = 0$ bypasses a supercurrent larger than other cases, presuming $I_{\rm s,m} > 0$. It is easy to derive the phase difference for the critical supercurrent

$$\frac{\partial I(\theta)}{\partial\theta} = 0 \rightarrow I_{\rm s}\cos\theta_{\rm c} + \frac{I_{\rm m}}{2}\cos\frac{\theta_{\rm c}}{2} = 0, \tag{S12}$$

which gives

$$\theta_{\rm c} = 2\arccos\left[\sqrt{\left(\frac{I_{\rm m}}{4I_{\rm s}}\right)^2 + \frac{1}{2}} - \frac{I_{\rm m}}{4I_{\rm s}}\right]. \tag{S13}$$

With $\zeta = \sqrt{(I_{\rm m}/4I_{\rm s})^2 + 1/2} - I_{\rm m}/4I_{\rm s}$, one obtains the critical current

$$I_c = (2I_{\rm s}\zeta + I_{\rm m})\sqrt{1-\zeta^2}. \tag{S14}$$

In the present scheme, when the current injected through the junction is larger than the critical current $I_{\rm c}$, a voltage drop is induced at the junction and drives the time evolution of superconducting phase difference according to the ac Josephson effect[6,7]. Conventionally

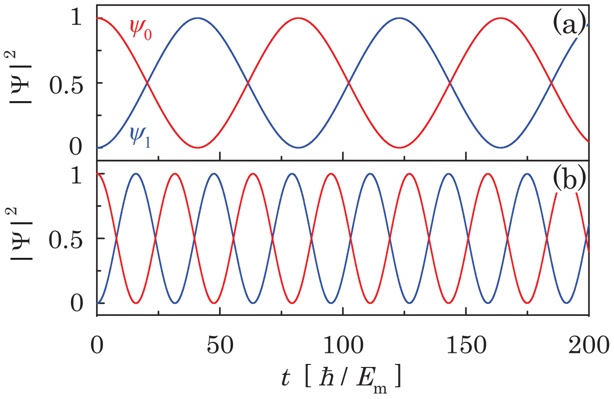

FIG. S1. (Color online). LZS interference between the two parity states upon current bias obtained by solving simultaneously Eqs. (6) in the main text and (S10) with $R = 5\hbar/2e^2$, $C = 8e^3/\hbar I_\mathrm{s}$, $I_\mathrm{m} = 0.5I_\mathrm{s}$ for which $I_\mathrm{c} \approx 1.375I_\mathrm{s}$, $I_\mathrm{ext} = 3I_\mathrm{s}$, and (a) $\delta/E_\mathrm{m} = 0.04$ and (b) $\delta/E_\mathrm{m} = 0.1$.

the RCSJ dynamics of the superconducting phase difference mimics a Newtonian particle moving in a tilted washboard potential[2,3]. In the present case, the phase particle acquires an additional pseudo-spin degree of freedom associated with the Majorana parity states as described by Eq. (S10).

The LZS oscillation of the Majorana qubit changes the supercurrent through the last term in Eq. (S10), and then recoils to influence the time evolution of superconducting phase difference. Therefore, the full dynamics of the system obey simultaneously the Schrödinger equation (6) in the main text and the RCSJ dynamics (S10). As shown in Fig. S1, for a bias current $I_\mathrm{ext} = 3I_\mathrm{s}$ corresponding to $\omega \simeq 30E_\mathrm{m}/\hbar$, the time evolutions of Majorana qubit derived with the RCSJ dynamics and those obtained under constant bias voltages shown in Fig. 4 in the main text agree well with each other. Therefore, one can conclude that the LZS interferometry for the Majorana qubit can be implemented in terms of moderate bias current through a junction with a small critical supercurrent. Here we note that, despite the injected current is larger than the critical current, the induced voltage can still be reduced to a value below the superconducting gap for appropriate device parameters.

In the RCSJ model, the injected current into the system is fixed, which then induces a voltage drop at the junction. The voltage oscillations with time can be obtained by solving numerically the Schrödinger equation (6) in the main text and the RCSJ dynamics (S10). As shown in Figs. S2(a) and (b), in contrary to the slow rotation of the Majorana qubit due

to the LZS interference, the voltage at the junction, which is nothing but the time derivative of the superconducting phase difference according to the ac Josephson relation[6,7], oscillates with time quickly. Two typical patterns in the voltage oscillation in Fig. S2(b) are displayed in Figs. S2(c) and (d), one associated with polarized qubit states (*i.e.* $|\psi_0| \gg |\psi_1|$ or vice versa) and the other with superposition states (*i.e.* $|\psi_0| \simeq |\psi_1|$), both described well by the potential function $I_\mathrm{s}\cos\theta + I_\mathrm{m}(|\psi_0|^2 - \psi_1|^2)\cos(\theta/2)$ as expected for an underdamped Josephson junction.

The full dynamics can be understood as follows. Taking into account the LZS oscillation of the Majorana qubit given in Eq. (7) in the main text, the RCSJ equation (S10) is reduced to

$$I_\mathrm{ext} = \frac{\hbar C\ddot{\theta}}{2e} + \frac{\hbar\dot{\theta}}{2eR} + I_\mathrm{s}\sin\theta + I_\mathrm{m}\cos(2\omega_\mathrm{m}t)\sin\frac{\theta}{2}. \tag{S15}$$

As an ansatz solution capturing the fundamental modes we presume

$$\theta(t) \simeq \omega t + A\sin(\omega t + \phi_1) + B\sin\left[(\frac{\omega}{2} + 2\omega_\mathrm{m})t + \phi_2\right] + B'\sin\left[(\frac{\omega}{2} - 2\omega_\mathrm{m})t + \phi_2'\right], \tag{S16}$$

where A, B, B', ω, ϕ_1, ϕ_2 and ϕ_2' are parameters to be determined. Plugging it into the RCSJ equation (S15), we encounter double-sine functions such as

$$\sin\theta = \sin\left(\omega t + A\sin(\omega t + \phi_1) + B\sin\left[(\frac{\omega}{2} + \omega_M)t + \phi_2\right] + B'\sin\left[(\frac{\omega}{2} - \omega_M)t + \phi_2'\right]\right). \tag{S17}$$

With the Jacobi-Anger expansion and checking the static term and those depending on time $\sin\omega t$, $\cos\omega t$, $\sin(\frac{\omega}{2} - \omega_M)t$, $\cos(\frac{\omega}{2} - \omega_M)t$, $\sin(\frac{\omega}{2} + \omega_M)t$ and $\cos(\frac{\omega}{2} + \omega_M)t$, we obtain the

parameters in the ansatz solution[3],

$$\begin{aligned}
\omega &= 2eRI_{\rm ext}/\hbar \\
\phi_1 &= \arctan[\frac{1}{Q(\omega/\omega_{\rm p})}] \\
A &= \frac{1}{(\omega/\omega_{\rm p})\sqrt{(\omega/\omega_{\rm p})^2+1/Q^2}} \\
\phi_2 &= \arctan[\frac{1}{Q(\omega/2+2\omega_{\rm m})/\omega_{\rm p}}] \\
B &= \frac{I_{\rm m}/2I_{\rm s}}{[(\omega/2+2\omega_{\rm m})/\omega_{\rm p}]\sqrt{[(\omega/2+2\omega_{\rm m})/\omega_{\rm p}]^2+1/Q^2}} \\
\phi_2' &= \arctan[\frac{1}{Q(\omega/2-2\omega_{\rm m})/\omega_{\rm p}}] \\
B' &= \frac{I_{\rm m}/2I_{\rm s}}{[(\omega/2-2\omega_{\rm m})/\omega_{\rm p}]\sqrt{[(\omega/2-2\omega_{\rm m})/\omega_{\rm p}]^2+1/Q^2}},
\end{aligned} \tag{S18}$$

where $\omega_{\rm p} = \sqrt{2eI_{\rm s}/\hbar C}$ is the plasma frequency and $Q = \sqrt{2eI_{\rm s}R^2C/\hbar}$ is the quality factor[2].

The voltage drop at the junction is given by the time derivative of the phase difference $V(t) = \frac{\hbar}{2e}\frac{d\theta(t)}{dt}$. Accordingly, the time averaged voltage is given by $\overline{V} = \omega\hbar/2e$, and the oscillating part of the voltage is characterized by a spectrum function which is the Fourier transformation

$$V(f) = \frac{1}{T}\int_0^T e^{-ift}[V(t)-\bar{V}]dt = A\delta(f-\omega) + B\delta(f-\frac{\omega}{2}-2\omega_{\rm m}) + B'\delta(f-\frac{\omega}{2}+2\omega_{\rm m}). \tag{S19}$$

The power spectrum of the voltage oscillation evaluated by the Fourier transformation on data in Fig. S2(b) is displayed in Fig. S2(e), which agrees with our analytical results quite well. The following two features are noticed here: (1) there are spectrum weights around $f = e\overline{V}/\hbar$, corresponding to 4π-period oscillations, due to the contribution from MQs [see Eq. (S10)], in addition to the peak at $f = 2e\overline{V}/\hbar$ according to the conventional ac Josephson relation; (2) there is a beat in the voltage oscillation and correspondingly a splitting in the spectrum peaks shown in the inset of Fig. S2(e) associated with the rotation of Majorana qubit given in Eq. (7) in the main text, with the typical frequency difference proportional to the MQ interaction δ. Because the oscillation of voltage at the junction under current bias is accompanied by a microwave radiation[2,3], the spectrum in Fig. S2(e) can be used for monitoring the quantum mechanical rotation of the Majorana qubit associated with the LZS interference.

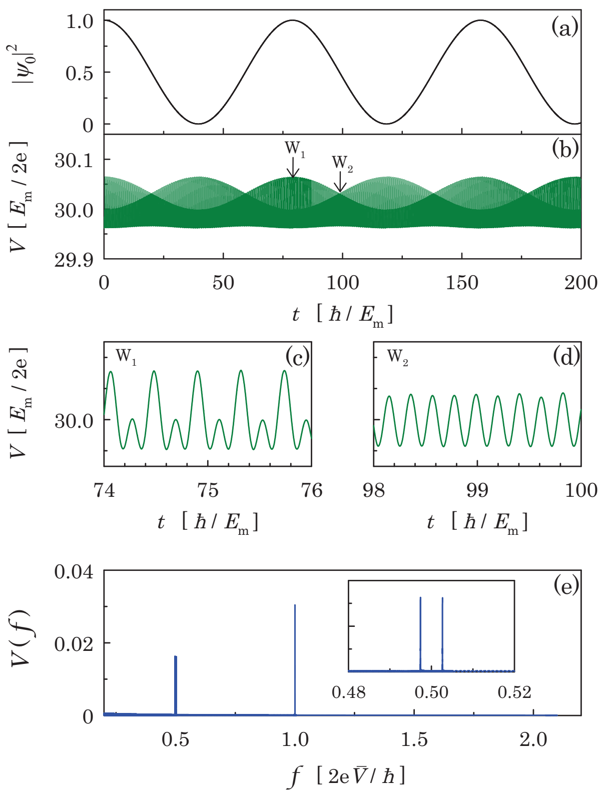

FIG. S2. (a) Time evolution of the Majorana qubit state, (b) that of the voltage across the tunneling junction, (c) and (d) typical patterns of voltage oscillations at the time windows marked in (b), and (e) spectrum of the voltage; inset: the fine structure around $f = e\overline{V}/\hbar$. Parameters are the same as Fig. S1(a).

There are two time scales in Eq. (S10), the Josephson oscillation time $\tau_{\rm J} = \phi_0/2\pi I_{\rm s} R$, and the damping time $\tau_{\rm RC} = RC$, yielding the Steward-McCumber number $\beta_{\rm C} = \tau_{\rm RC}/\tau_{\rm J}$ related to the quality factor of the Josephson oscillation in the junction[2]. For the parameters in Fig. S1, one has $\beta_{\rm C} = 100 \gg 1$, corresponding to a highly underdamped junction. In this case, decoherence of the LZS interference cannot be found over sufficiently long time as shown in Fig. S3. In a highly underdamped Josephson junction there is a hysteresis loop in the I-V characteristics, and a phase dynamic state with a finite voltage drop across the junction is possible even though the current injection is below the critical current[3], which may also be used for achieving the LZS interferometry for Majorana qubit.

In the above discussions, we have presumed a pure Majorana qubit state (even- or odd-parity state) as the initial condition of quantum mechanical evolutions. As read from E-

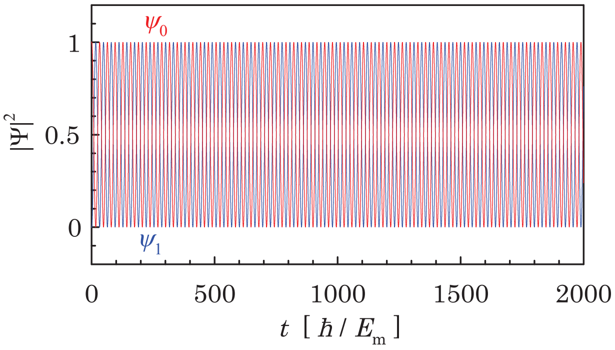

FIG. S3. LZS oscillations of the Majorana qubit over a long time. Parameters are the same as Fig. S1(a).

q. (S10), the maximal supercurrent in the parallel circuit of Cooper-pair channel and MQ channel is achieved by a pure state. This feature can be exploited to initialize the qubit into a pure state by increasing gradually the current injection up to the maximal supercurrent.

In a very recent experiment, rf radiations were detected from a Josephson junction in the heterostructure of the topological insulator HgTe and an s-wave superconductor, a candidate system for realizing topological superconductivity[8]. A half-frequency component with broad line width was observed in addition to the conventional Josephson one, which may be relevant to the physics discussed in the present work. It is anticipated that experiments with finer frequency resolution based on our protocol will reveal the quantum mechanical dynamics of MQ, and pave the way for building a universal gate for Majorana qubit.

To finish this section we notice that the current injected into the system beyond the critical current triggers a large voltage drop across the junction, which then may induce undesired coupling between the MQs and quasiparticles in the continuum spectrum. In this sense, the voltage bias is a better implementation of the LZS interferometry for the Majorana qubit.

Typical values of physical quantities. Finally we give typical numbers for relevant physical quantities. The topological superconducting gap is in the order of 200 μeV according to the state-of-art experiments[9,10], whereas the coupling of MQs at the junction should be smaller by one order or so, say $E_{\rm m} = 10$ μeV. The coupling between MQs in individual 1D topological superconductors, the smallest energy scale of the present system, is $\delta = 5$ μeV.

Typical critical current is of $I_\mathrm{s} = 10$ nA according to experimental results in nanowire junctions[9], which gives the order of bias current. Typical shunted resistance and capacitance are of $R = 5\hbar/2e^2 = 10.3$ kΩ and $C = 8e^3/\hbar I_\mathrm{s} = 0.03$ pF. LZS frequency in a system with the above parameter is $\omega_\mathrm{m} = 1.3$ GHz according to our theory, when a current $I = 3I_\mathrm{s}$ is biased which raises a Josephson frequency $\omega = 76$ GHz. The Josephson oscillation time is $\tau_\mathrm{J} = \phi_0/2\pi I_\mathrm{s} R = 3$ ps, and the damping time is $\tau_\mathrm{RC} = RC = 0.3$ ns. The Joule heating is approximately $V^2/R = 25fW$ for a voltage bias of $V = 50\mu V$, which is small enough and will not damage the function of the device.

---

1. Son, S. K., Han, S. & Chu, S. I. Floquet formulation for the investigation of multiphoton quantum interference in a superconducting qubit driven by a strong ac field. *Phys. Rev. A.* **79**, 032301 (2009).
2. Tinkham, M. Introduction to Superconductivity, 2nd edn (Dover Publications, 1996).
3. Hu, X. & Lin, S. Z. Phase dynamics in a stack of inductively coupled intrinsic Josephson junctions and terahertz electromagnetic radiation. *Supercond. Sci. Technol.* **23**, 053001 (2010).
4. Kitaev, A. Unpaired Majorana fermions in quantum wires. *Phys.-Usp.* **44**, 131-136 (2001).
5. Alicea, J., Oreg, Y., Refael, G., von Oppen, F. & Fisher, M. P. A. Non-Abelian statistics and topological quantum information processing in 1D wire networks. *Nature Phys.* **7**, 412-417 (2011).
6. Josephson, B. D. Possible new effects in superconductive tunnelling. *Phys. Lett.* **1**, 251 (1962).
7. Josephson, B. D. Coupled Superconductors. *Rev. Mod. Phys.* **36**, 216 (1964).
8. Deacon, R. S. et al. Josephson radiation from gapless Andreev bound states in HgTe-based topological junctions. *Phys. Rev. X.* **7**, 021011 (2017).
9. Mourik, V. et al. Signatures of Majorana fermions in hybrid superconductor-semiconductor nanowire devices. *Science.* **336**, 1003-1007 (2012).
10. Albrecht, S. M. et.al. Exponential protection of zero modes in Majorana islands. *Nature.* **531**, 206 (2016).
